# The mitochondrial genome of the yellow-vented flowerpecker, *Dicaeum chrysorrheum* (Dicaeidae) from southwestern China

**DOI:** 10.1080/23802359.2021.1972483

**Published:** 2021-09-06

**Authors:** Yong Gao, Si Yin, Lei Zhu

**Affiliations:** College of Biological Resource and Food Engineering, Qujing Normal University, Qujing, China

**Keywords:** Yellow-vented flowerpecker, *Dicaeum chrysorrheum*, mitogenome, phylogenetic analysis

## Abstract

The yellow-vented flowerpecker (*Dicaeum chrysorrheum* Temminck & Laugier) is a small bird in the Dicaeidae family. To explore the phylogenetic relationships within the genus *Dicaeum*, we sequenced the complete mitochondrial genome of *D. chrysorrheum* using a next generation sequencing platform. The mitogenome of *D. chrysorrheum* (GenBank accession number, MW629121) has a length of 16,818 bp, with a nucleotide composition of 30.62% A, 23.45% T, 31.34% C, and 14.59% G. The genome is comprised of 13 protein-coding genes, 22 transfer RNA genes, and two ribosomal RNA genes. The total length of the protein-coding genes is 11,402 bp, accounting for 67.8% of the total length of the mitochondrial genome. Of the 13 protein-coding genes, 10 have ATG start codons and three genes terminate with incomplete stop codons. A maximum-likelihood phylogenetic tree of 16 bird species placed *D. chrysorrheum* and *D. agile* as sister to the other three *Dicaeum* species (*D. concolor*, *D. eximium* and *D. cruentatum*). This new mitogenome will be useful for further phylogenetic studies of the genus *Dicaeum*.

The yellow-vented flowerpecker (*Dicaeum chrysorrheum* Temminck & Laugier) is a passerine bird belonging to the Dicaeidae family (Cheke and Mann 2008). Adult *D. chrysorrheum* have olive green feathers on the top of their bodies and are white with black stripes on the underside. This bird usually lives in intermontane plains, alpine broad leaved forests, and bushes near farm lands (Cheke and Mann 2008). The yellow-vented flowerpeckers are very active, and they are often seen on treetops (Sheldon 1985). This species is a widely-distributed resident bird in many countries of East Asia and Southeast Asia, such as southwestern China (Yunnan and Guanxi provinces), Nepal, Sikkim, Bhutan, India, and Indonesia (Nyári et al. 2009).

In this study, we generated the full mitochondrial genome of *D. chrysorrheum* using a next generation sequencing (NGS) platform. The *D. chrysorrheum* sample was collected from Mengla (E 101°32′28″, N 21°27′34.2″), in Yunnan Province, China. The sample collection had been approved by the local administrative of forestry. Blood was taken from the brachial vein, and total genomic DNA was extracted using a commercial blood DNA isolation kit (DP318; TIANGEN, Beijing, China). The specimen and genomic DNA were stored in the herbarium of the College of Biological Resource and Food Engineering, Qujing Normal University (voucher number, QJNU-Zhu-20200804-Dc; Lizhou Tang, biologytang@163.com). The sequencing library was generated using the VAHTS Universal DNA Library Prep Kit (Vazyme, Nanjing, China), and 1 μg purified DNA. The library was quantified using a Qubit 3.0 fluorometer (Life Technologies, Carlsbad, CA, USA) and size selection was done with a Bioanalyzer 2100 (Agilent Technologies, CA, USA). Subsequently, DNA sequencing was performed on a MGI-SEQ 2000 platform by Frasergen Bioinformatics Co., Ltd. (Wuhan, China). Genome sequences were assembled using SPAdes 3.5.0 (http://cab.spbu.ru/software/spades/) (Bankevich et al. 2012). Mitogenomic features, such as protein-coding genes, tRNAs, and rRNAs were annotate with an online tool, GeSeq (http://www.ncbi.nlm.nih.gov/gorf/gorf.html) (Tillich et al. 2017), and then manually adjusted using the mitogenome of *D. eximium* (GenBank accession number: NC_051023.1) as a reference.

The complete mitogenome of *D. chrysorrheum* (GenBank accession number: MT985377) is 16,818 bp in length, including 13 protein-coding genes, 22 transfer RNA genes, and two ribosomal RNA genes. The GC content of the mitogenome is 45.93%, with a nucleotide composition of 30.62% A, 23.45% T, 31.34% C, and 14.59% G. The total length of the protein-coding genes is 11,402 bp, accounting for 67.8% of the total length of the mitochondrial genome. Of the 13 protein-coding genes, 10 have ATG start codons. However, ND2 and ND3 start with ATA, and COX1 starts with GTG. Three genes terminate with incomplete stop codons (ND2, COX3, and ND4), while the remaining 10 end with complete stop codons (AGG, TAA, TAG or AGA) (Table S1).

To infer the phylogenetic position of *D. chrysorrheum*, mitogenomes of 15 Passeriformes birds were downloaded from the NCBI nucleotide database. The mitogenome sequences were aligned with MEGA 7 (Kumar et al. 2016) using the ClustalW algorithm (Thompson et al. 2002). A phylogenetic tree was then constructed using MEGA 7 (Kumar et al. 2016) with the maximum likelihood (ML) method based on the general time reversible (GTR) model (Jukes and Cantor 1969), and 1000 bootstrap replicates were calculated to assess the robustness of the phylogenetic interference. The resulting phylogenetic tree showed that five *Dicaeum* species formed a monophyletic clade (Figure 1). The mitogenome of *D. chrysorrheum* provides genetic resources for future phylogenetic studies of the *Dicaeum* genus.

**Figure 1.**
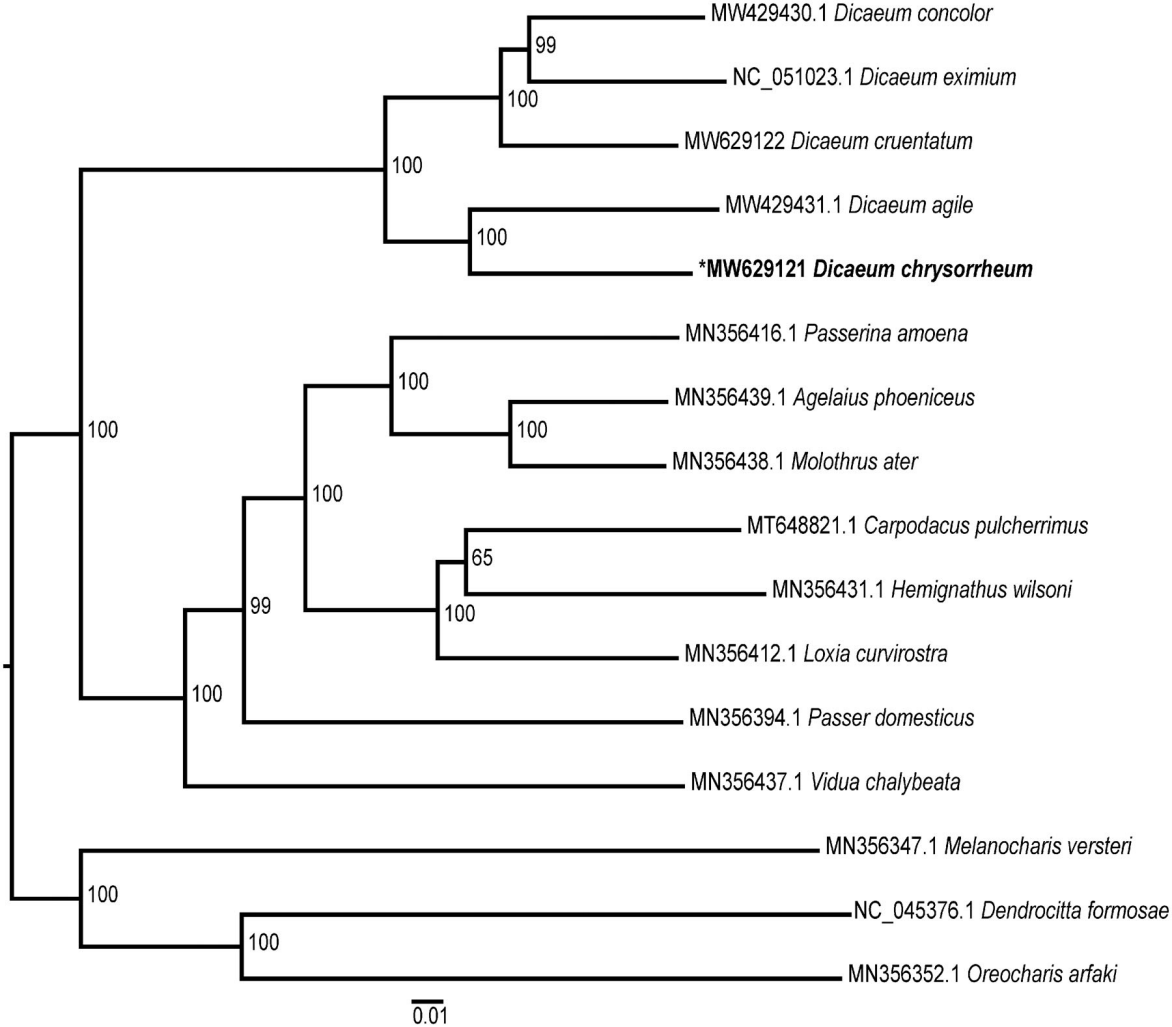
The maximum-likelihood (ML) tree constructed using mitochondrial genome sequences from *Dicaeum chrysorrheum* and 15 other Passeroidea species. Bootstrap values based on 1000 replicates are shown for each node.

## Supplementary Material

Supplemental Material

## Data Availability

The genome sequence data that support the findings of this study are openly available in GenBank of NCBI at (https://www.ncbi.nlm.nih.gov/) under the accession number MW629121. The associated BioProject, SRA, and Bio-Sample numbers are PRJNA732243, SRS9047724, and SAMN19314806 respectively.
