# Supplementary material for: The mitochondrial genome of the yellow-vented flowerpecker, Dicaeum chrysorrheum (Dicaeidae) from southwestern China
Source: Mitochondrial DNA B Resour. 2021 Sep 6;6(10):2860–2. doi: 10.1080/23802359.2021.1972483 (PMC8425760; doi:10.1080/23802359.2021.1972483)
Supplement: Supplemental Material [file TMDN_A_1972483_SM9486.pdf]

Table S1. Gene annotations of the yellow-vented flowerpecker mitogenome.

Gene ID	Location	Start codon	Stop codon	product
ND1	2803..3780	ATG	AGG	NADH-ubiquinone oxidoreductase chain 1
ND2	4008..5047	ATA	TA-	NADH-ubiquinone oxidoreductase chain 2
COX1	5405..6955	GTG	AGG	Cytochrome c oxidase subunit 1
COX2	7098..7781	ATG	TAA	Cytochrome c oxidase subunit 2
ATP8	7853..8020	ATG	TAA	ATP synthase F0 subunit 8
ATP6	8011..8694	ATG	TAA	ATP synthase F0 subunit 6
COX3	8701..9485	ATG	T-	cytochrome C oxidase subunit III
ND3	9554..9904	ATA	TAA	NADH-ubiquinone oxidoreductase chain 3
ND4L	9977..10273	ATG	TAA	NADH-ubiquinone oxidoreductase chain 4L
ND4	10267..11649	ATG	T-	NADH-ubiquinone oxidoreductase chain 4
ND5	11850..13667	ATG	AGA	NADH dehydrogenase subunit 5
CYTB	13677..14819	ATG	TAA	Cytochrome b
ND6	complement (15499..14981)	ATG	TAG	NADH-ubiquinone oxidoreductase chain 6
12S_rRNA	68..1053	-	-	12S ribosomal RNA
16S_rRNA	1123..2722	-	-	16S ribosomal RNA
trnF(gaa)	1..68	-	-	tRNA-Phe
trnV(uac)	1053..1122	-	-	tRNA-Val
trnL(uaa)	2723..2797	-	-	tRNA-Leu
trnI(gau)	3790..3861	-	-	tRNA-Ile
trnM(cau)	3939..4007	-	-	tRNA-Met
trnW(uca)	5048..5117	-	-	tRNA-Trp
trnD(guc)	7023..7091	-	-	tRNA-Asp
trnK(uuu)	7783..7851	-	-	tRNA-Lys
trnG(ucc)	9485..9553	-	-	tRNA-Gly
trnR(ucg)	9906..9975	-	-	tRNA-Arg
trnH(gug)	11645..11714	-	-	tRNA-His
trnS(gcu)	11715..11779	-	-	tRNA-Ser
trnL(uag)	11779..11849	-	-	tRNA-Leu
trnT(ugu)	14823..14892	-	-	tRNA-Thr
trnE(uuc)	complement (15572..15501)	-	-	tRNA-Glu
trnP(ugg)	complement (14970..14901)	-	-	tRNA-Pro
trnS(uga)	complement (7019..6947)	-	-	tRNA-Ser
trnY(gua)	complement (5403..5334)	-	-	tRNA-Tyr
trnC(gca)	complement (5333..5267)	-	-	tRNA-Cys
trnN(guu)	complement (5266..5194)	-	-	tRNA-Asn
trnA(ugc)	complement (5187..5119)	-	-	tRNA-Ala
trnQ(uug)	complement (3939..3869)	-	-	tRNA-Gln

For Peer Review Only
